# Supplementary figures and images for: Examining the role of the surfactant family member SFTA3 in interneuron specification
Source: PLoS One. 2018 Nov 8;13(11):e0198703. doi: 10.1371/journal.pone.0198703 (PMC6224035; doi:10.1371/journal.pone.0198703)

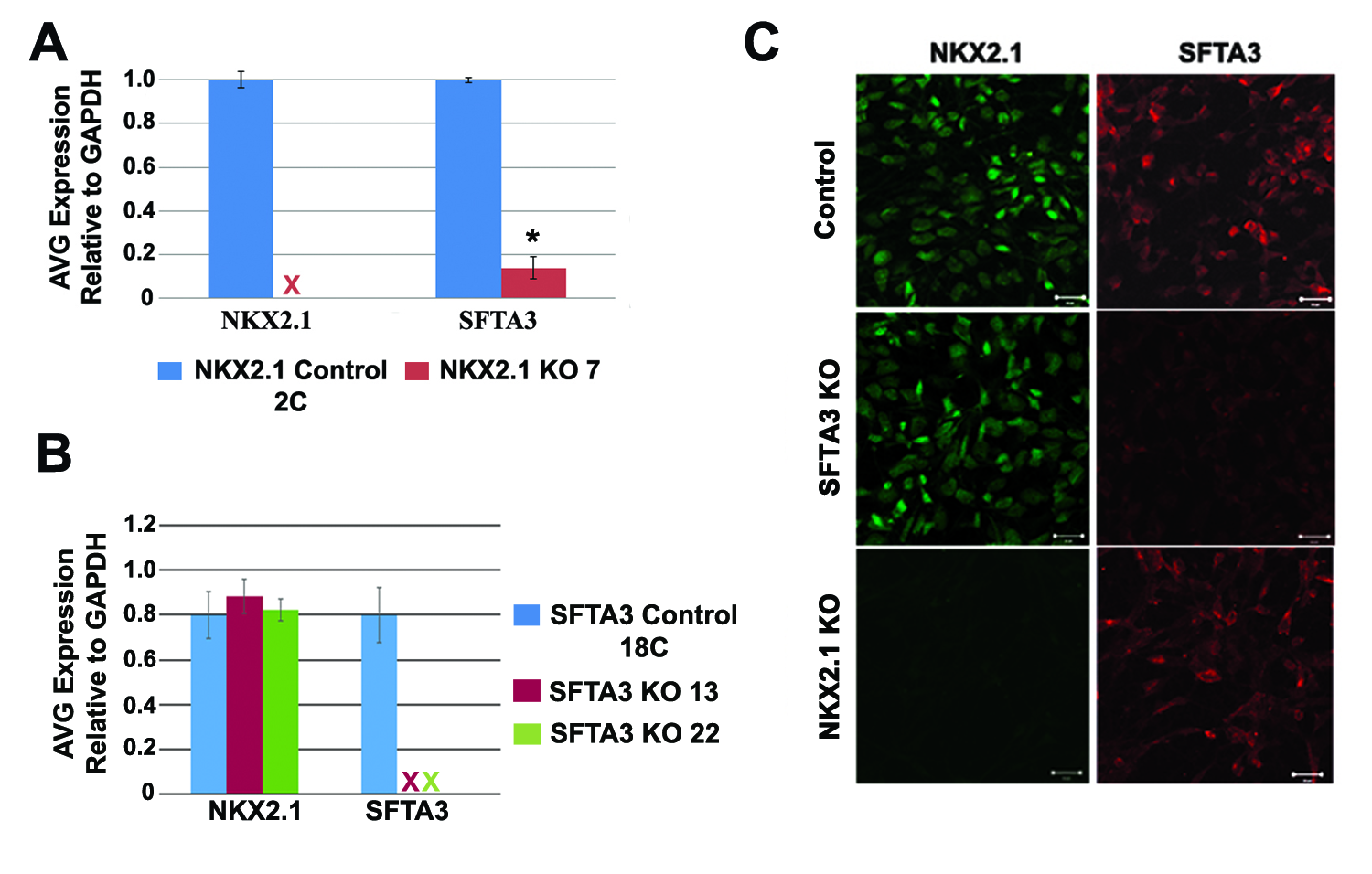

Supplement: S1 Fig — A) qRT-PCR data comparing NKX2.1 and SFTA3 expression levels between day 25 NKX2.1 control and KO cell progenitors. Data represented as mean ± SEM. * = p<0.05. B) qRT-PCR data comparing NKX2.1 and SFTA3 gene expression between day 25 SFTA3 control and KO cell progenitors. Data represented as mean ± SEM. * = p<0.05. C) Day 25 immunocytochemistry analysis of NKX2.1 and SFTA3 knockout and control hESNPs. Scale bar = 20 μm. (TIF) [file pone.0198703.s001.tif]

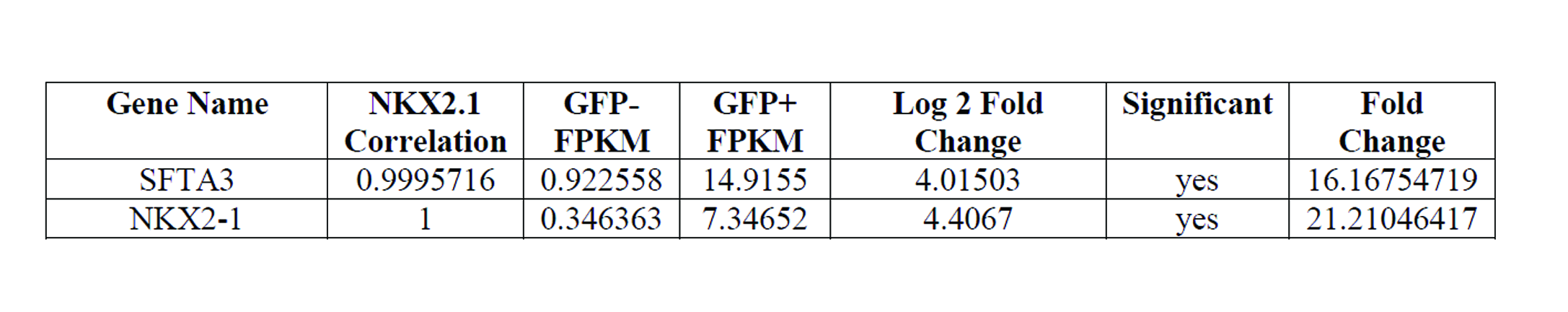

Supplement: S1 Table — (TIF) [file pone.0198703.s002.tif]
